# Regeneration Potential of Jellyfish: Cellular Mechanisms and Molecular Insights

**DOI:** 10.3390/genes12050758

**Published:** 2021-05-17

**Authors:** Sosuke Fujita, Erina Kuranaga, Yu-ichiro Nakajima

**Affiliations:** 1 Graduate School of Life Sciences, Tohoku University, Sendai 980-8578, Miyagi, Japan; sosuke.rinama@gmail.com (S.F.); erina.kuranaga.d1@tohoku.ac.jp (E.K.); 2 Frontier Research Institute for Interdisciplinary Sciences, Tohoku University, Sendai 980-8577, Miyagi, Japan

**Keywords:** jellyfish, medusa, regeneration, radial symmetry, wound healing, stem/progenitor cells, transdifferentiation, dedifferentiation, patterning

## Abstract

Medusozoans, the Cnidarian subphylum, have multiple life stages including sessile polyps and free-swimming medusae or jellyfish, which are typically bell-shaped gelatinous zooplanktons that exhibit diverse morphologies. Despite having a relatively complex body structure with well-developed muscles and nervous systems, the adult medusa stage maintains a high regenerative ability that enables organ regeneration as well as whole body reconstitution from the part of the body. This remarkable regeneration potential of jellyfish has long been acknowledged in different species; however, recent studies have begun dissecting the exact processes underpinning regeneration events. In this article, we introduce the current understanding of regeneration mechanisms in medusae, particularly focusing on cellular behaviors during regeneration such as wound healing, blastema formation by stem/progenitor cells or cell fate plasticity, and the organism-level patterning that restores radial symmetry. We also discuss putative molecular mechanisms involved in regeneration processes and introduce a variety of novel model jellyfish species in the effort to understand common principles and diverse mechanisms underlying the regeneration of complex organs and the entire body.

## 1. Introduction

**Figure 1:**
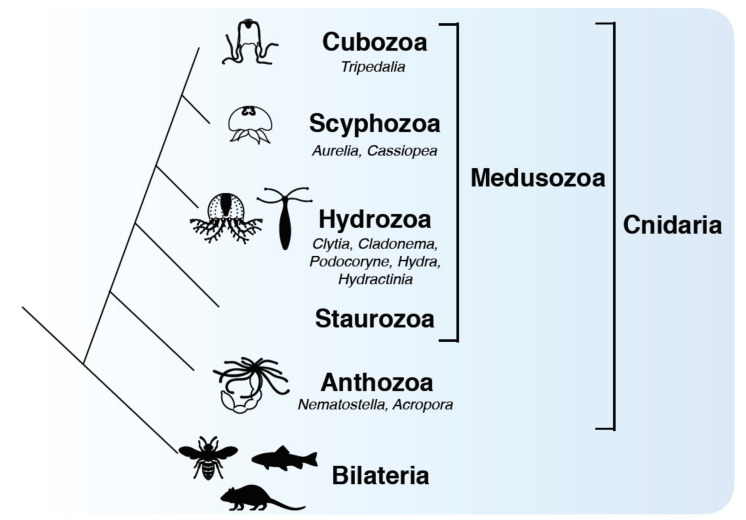
The phylogenetic position of medusozoans in the animal kingdom. Cnidaria is the sister group of Bilateria and includes Anthozoa and Medusozoa. Jellyfish, characterized by the exhibition of a medusa stage, belong to Medusozoa, which consists of classes Hydrozoa, Scyphozoa, Cubozoa, and Staurozoa.

As the sister group of bilaterians, cnidarians, including jellyfish, are one of the oldest groups of animals possessing a nervous system and muscles [1]. Cnidarians are aquatic animals, and most of them live in marine environments. The first cnidarians appeared on the earth an estimated 600 million years ago and have evolved in response to a variety of environmental stimuli. The phylum Cnidaria is classified into two clades: Anthozoa and Medusozoa. While Anthozoa includes corals and sea anemones, Medusozoa includes the four classes Hydrozoa, Scyphozoa, Cubozoa, and Staurozoa, which are all commonly called jellyfish, exhibiting a medusa phase (with the exception of some hydroids) (Figure 1) [2]. As cnidarians, jellyfish have the following common features: (i) a diploblastic body consisting of ectoderm and endoderm with radial symmetry; (ii) stinging cells, or cnidocytes (nematocytes), with a specialized cellular organelle called the cnidocyst (nematocyst), which contains a toxin for predation and defense; (iii) a high regenerative ability to restore lost organs and nearly the entire body [3,4,5]. Despite possessing cnidarian-specific common characteristics, jellyfish morphologies including shape, size, and colors, have diversified among different classes and within the same class [6].

**Figure 2:**
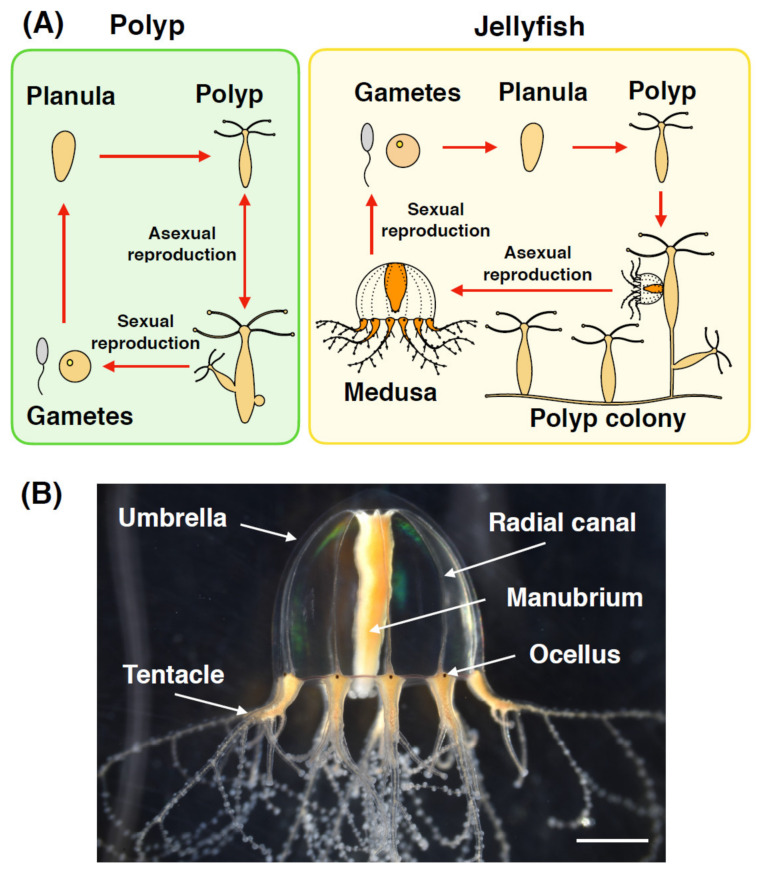
Life cycle and morphology of jellyfish. (**A**) The life cycle of the cnidarian polyp and jellyfish, representative of Hydrozoa. In the polyp type life cycle, a polyp reproduces both asexually and sexually. In the jellyfish life cycle, polyps asexually give rise to medusae, and medusae produce gametes for sexual reproduction. (**B**) Picture of *Cladonema* medusa as a representative hydrozoan jellyfish. The medusa is comprised of the umbrella, manubrium, radial canals, and tentacles including ocellus at the bulb region. Scale bar, 1.0 mm.

Most jellyfish take two main distinct forms in their life cycle: sessile polyps and free-swimming medusae. After the fertilization of gametes, zygotes develop into planula larvae, which metamorphose into polyps, and mature polyps give rise to medusae through a process of budding or strobilation (Figure 2A). Like other polyp-type cnidarians, polyps grow clonally by asexual reproduction, while medusae perform sexual reproduction. In its essence, the bell-shaped body of a medusa is composed of the following organs: an umbrella for swimming, a manubrium for feeding and digestion, gonads for reproduction, tentacles for capturing prey, and radial canals for nutrient transportation throughout the body (Figure 2B). To control these various organs, medusae have evolved multiple types of muscles (smooth and striated) and an intricate nervous system that include sensory organs with functional eyes (rhopalia) and balance organs (statocysts) [3,7].

Despite their relatively complex morphology compared to polyps, medusae are capable of regenerating various organs and even reconstituting their entire body from fragments [8,9,10,11,12,13,14,15]. Classical in vitro experiments have also shown that isolated muscle from the medusae umbrella can reconstruct de novo organs under the special culture conditions [16]. Furthermore, in some jellyfish, including the “immortal jellyfish”, *Turritopsis* *dohrnii*, adult medusae can transform into cysts after injury or starvation and eventually return to polyps, a phenomenon known as “reverse development” [17,18]. These observations indicate that the medusa stage exhibits a high regenerative capacity, which likely varies across different species. While cnidarian model polyps such as *Hydra*, *Hydractinia* and *Nematostella* have been powerful models for understanding animal regeneration, jellyfish species constitute a unique model to investigate the common principles and diverse mechanisms underlying the regeneration of complex organs and the whole body.

So, what are the mechanisms that enable regeneration in jellyfish? Although the varied regenerative capacity of medusae has long been documented, recent studies have only just begun to elucidate the detailed cellular mechanisms that underlie it. Currently, due to the lack of genetic manipulations in most jellyfish species, the molecular understanding of regeneration processes is quite limited. In this review, we will explore the cellular processes involved in medusa regeneration, including wound healing, stem/progenitor cell proliferation, and trans/dedifferentiation, and will speculate on the molecular mechanisms associated with each process. We will also focus on the whole-body regeneration potential of medusae by highlighting in particular the patterning events during reconstitution of radial symmetry. Lastly, we will discuss future directions of research in jellyfish regeneration by sharing unanswered questions and introducing novel model jellyfish species.

## 2. Wound Healing

Wound healing is an initial process of regeneration that accompanies tissue remodeling in order to close physical gaps and restore tissue integrity after damage. Accurate wound healing plays an important role in maintaining the homeostasis of the internal environment and preventing infection [19]. In epithelial contexts, wound healing consists of several cellular processes including cell migration, cell proliferation, and cell differentiation [20]. Upon tissue injury, cell migration first occurs with cells moving toward the damage site, which necessitates actin cytoskeleton reorganization and membrane protrusion formation such as lamellipodia [21]. Subsequently, cell proliferation and cell differentiation take place, restoring epithelial architecture. Given that diploblastic cnidarians are formed mainly by epithelial cells, wound healing programs must fall under the umbrella of regeneration processes of medusae.

**Figure 3:**
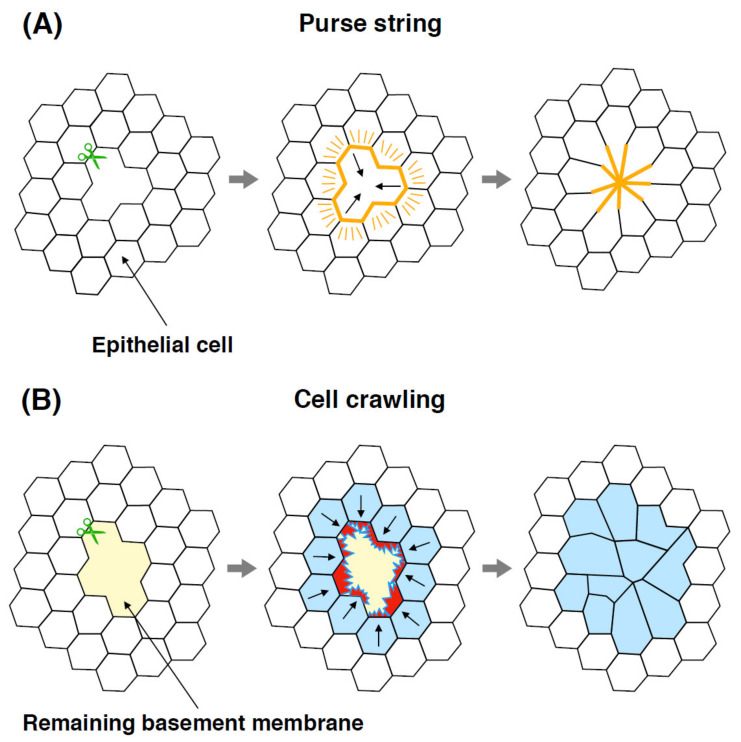
Schematic of two different cellular processes of wound healing. (**A**) During purse string healing, supracellular actomyosin cables (orange lines) accumulate around the wound circumference, and the cells at the wound periphery contract. (**B**) During cell crawling, lamellipodia-dependent cell migration covers the wound gap, and the migrating cells close the wound. Red protrusions indicate lamellipodia.

Using the hydrozoan jellyfish *Clytia hemisphaerica*, cellular mechanisms of wound healing have been investigated by establishing live imaging protocols. The medusa umbrella, or bell, is composed of ectodermal epithelial cells that are roughly hexagonal in shape, mesoglea, and two types of muscle cells. When the exumbrella epithelial sheet, the outer surface of the bell, is injured, the wound immediately closes via two cellular processes: purse string healing and lamellipodia-dependent cell crawling [22]. The purse string mechanism functions as a small-circular wound closure where actin and myosin II form a supracellular cable around the wound circumference (Figure 3A). By contrast, cell crawling utilizes lamellipodia-dependent cell migration to cover the gap and involves steps of zippering, contraction, and relaxation, contributing to relatively large-sized wound closure (Figure 3B). Drug treatment by blebbistatin, an inhibitor of myosin II, suppresses the process of cell contraction, suggesting that actomyosin contractility is necessary for proper wound closure in the umbrella epithelium. While cell crawling occurs as long as the basement membrane is intact, the purse string mechanism alternatively dominates if the basement membrane is disrupted, implicating a switch-like mechanism of wound healing in response to surrounding environments [22]. Cell migration with lamellipodia formation is also reported during wound healing of the medusae umbrella in *Polyorchis penicillatus,* a different species of hydrozoan jellyfish [23]. Of note, wound closure in *Clytia* and *Polyorchis* umbrellas does not require cell proliferation [10,22,23]. As has been suggested in other animal systems, the selection of purse string or cell crawling action depends on wound size, geometry, and tissue type [21,24,25]. The involvement of actin cytoskeleton and the lack of cell proliferation in wound closure are also observed during wound healing in mammalian cell cultures and *Drosophila* larvae [26,27,28,29]. These results indicate that cellular mechanisms of wound healing share an evolutionary origin and are likely conserved throughout the animal kingdom.

Despite the similarity in cellular dynamics across different organisms, the molecular mechanisms that coordinate the processes of wound healing in jellyfish remain elusive. MAPK signaling, including ERK, JNK, and p38, which plays a role in many animals’ regeneration, is a likely candidate to regulate wound healing in basal metazoans. Indeed, in anthozoan *Nematostella vectensis*, a polyp type cnidarian, ERK signaling is crucial for proper wound closure and the upregulation of downstream signaling associated with regeneration [30]. By contrast, during head regeneration of *Hydra*, a hydrozoan polyp, ERK, JNK, and p38 are not required for wound healing, but their signaling is essential for subsequent head regeneration [31]. It will be interesting to clarify the roles of MAPK signaling during wound healing of medusae, which may lead to a better understanding of conserved or diversified molecular mechanisms underlying epithelial wound healing.

## 3. Stem/Progenitor Cell Proliferation

After wound closure, the next step in regeneration often requires the production of new cells, or blastema, to reconstruct lost structures. The regeneration blastema can be generated by simply increasing the number of undifferentiated cells by tissue-resident stem/progenitor cell proliferation. Depending on their potency (toti-/pluri-/multi-potent), stem/progenitor cells are able to differentiate into any or specialized cell types while having the ability to self-renew [32]. For example, animals with a highly regenerative capacity such as planarians possess pluripotent stem cells, called neoblasts, which allow regeneration of the whole body from small fragments. Similarly, cnidarians have undifferentiated stem or stem-like cells such as interstitial cells (i-cells) in hydrozoans and amebocytes in non-hydrozoans [33]. In particular, studies using *Hydra* and *Hydractinia* have revealed that hydrozoan i-cells can produce both somatic and germ cell types, and have identified molecular markers of i-cells including *Piwi*, *Nanos*, and *Vasa*, as well as their proliferative and migratory capacities [34,35,36,37,38,39,40,41]. Indeed, cell proliferation mediated by stem/progenitor cells is necessary for *Hydra* basal head regeneration, *Nematostella* oral regeneration, and *Hydractinia* head regeneration [42,43,44].

**Figure 4:**
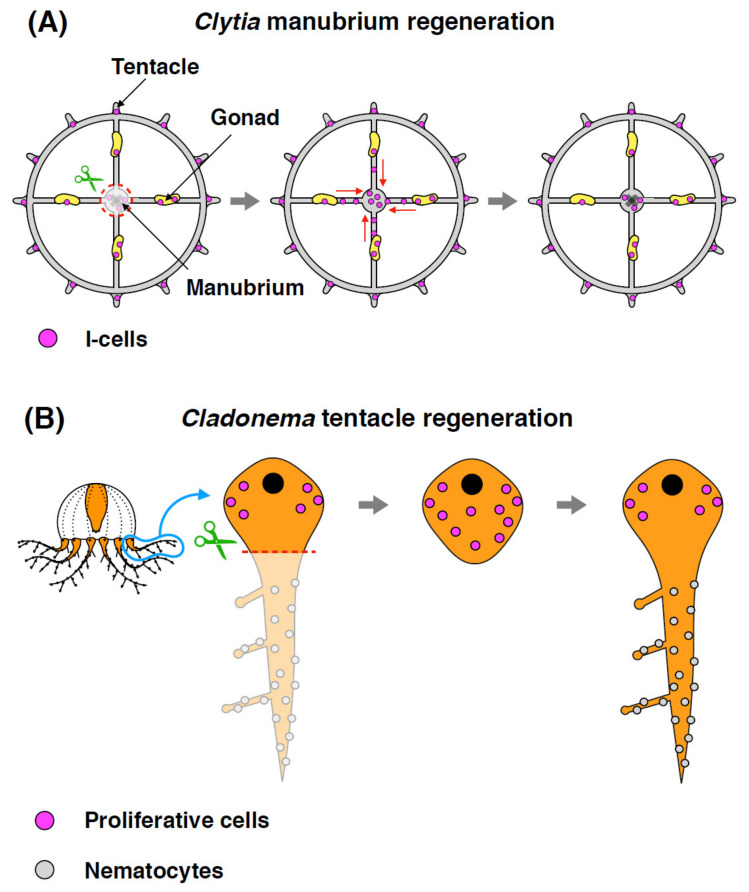
Schematic depicting jellyfish organ regeneration. (**A**) During *Clytia* manubrium regeneration, i-cells proliferate and migrate from the gonads or tentacles to the regenerating area. (**B**) After tentacle amputation in *Cladonema*, the number of proliferative cells increase in response to the injury and contribute to tentacle regeneration.

Although it is currently unclear whether functional stem cells exit in jellyfish, *Clytia* medusae possess i-cells that are defined by their large nuclear-to-cytoplasmic ratios and conserved molecular markers, which are localized in the tentacle bulbs, gonads, and manubrium [45]. Sinigaglia et al. recently reported stem cell dynamics during the *Clytia* manubrium regeneration [10]: when the original manubrium is completely removed, a new functional manubrium can regenerate within four days. The manubrium regeneration process requires cell proliferation, which was detected in quantity at 24 h post-dissection (hpd). The same work also demonstrated that i-cells in gonads can migrate toward the wound area and participate in the regenerating manubrium (Figure 4A) [10]. The timing of massive cell proliferation coincides with the time when i-cells are recruited, suggesting the possibility that stem cell proliferation contributes to manubrium regeneration. In addition to the feeding manubrium, medusae can regenerate other organs such as tentacles and gonads [10,11]. Another study using the hydrozoan *Cladonema pacificum* showed that cell proliferation actively occurs at 24 hpd in tentacle bulbs, which are required for proper tentacle regeneration (Figure 4B) [11]. Other hydrozoan jellyfish, *Cytaeis uchidae* and *Rathkea octopunctata* also have proliferative cells in their tentacle bulbs and manubrium, suggesting the existence of resident stem-like cells in specific compartments [11]. Taken together, these results indicate that stem/progenitor cell proliferation is a critical factor for medusae organ regeneration.

How is stem cell proliferation controlled during jellyfish regeneration? The conserved Wnt/β-catenin signaling plays an important role in the maintenance of stem cell self-renewal as well as regeneration across different taxa [46,47,48,49]. Indeed, during *Hydra* basal head regeneration, β-catenin activation by Wnt3 induces cell proliferation of neighboring stem/progenitor cells [42]. Similarly, during *Clytia* manubrium regeneration, Wnt-pathway components and downstream targets were upregulated around the wound site [10]. In addition, inhibitor treatment for Wnt/β-catenin signaling prevented manubrium regeneration before blastema formation, suggesting a possibility that Wnt/β-catenin signaling controls stem/progenitor cell behaviors during medusa organ regeneration.

## 4. Transdifferentiation and Dedifferentiation

Although stem/progenitor cells play an important role in regeneration by providing a source of blastema, some animals exhibit regenerative abilities without a clear contribution by tissue-resident proliferating cells. When this is the case, regenerative blastema can be produced through the processes of transdifferentiation and/or dedifferentiation from mature differentiated cells. For example, during newt lens regeneration, pigment epithelial cells from the dorsal iris transdifferentiate to new lens cells and reconstitute the complete lens [50]. In the regenerating zebrafish heart, differentiated cardiomyocytes undergo dedifferentiation and proliferate as new cardiomyocytes that compensate for lost parts [51]. While transdifferentiation and dedifferentiation are mainly observed in vertebrate organ regeneration, cnidarian *Hydra* possess both stem-cell based mechanisms and transdifferentiation [52], suggesting that cell fate plasticity, together with proliferating stem/progenitor cells, reinforces their remarkable regenerative capacity. It is noteworthy that, compared to cnidarian polyps, stem/progenitor cells appear to be restricted to specific organs or compartments in medusae, raising the hypothesis that cell fate is more plastic in jellyfish.

**Figure 5:**
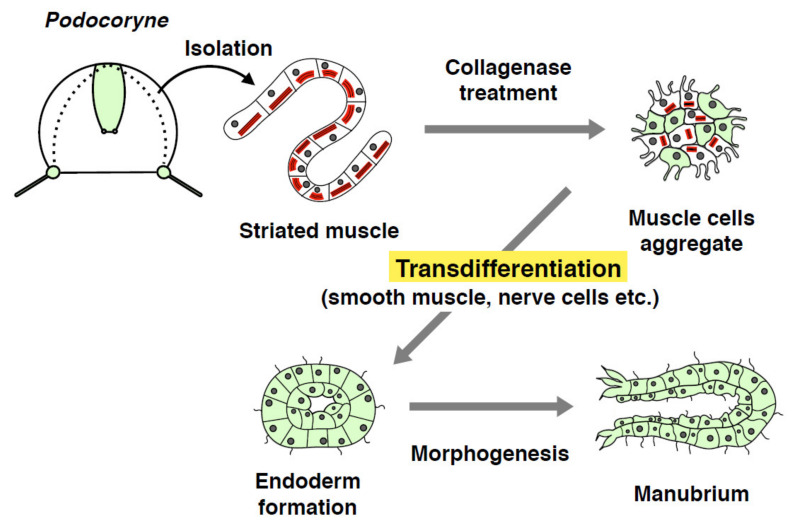
Schematic of in vitro transdifferentiation potential in the jellyfish *Podocoryne*. After collagenase treatment to isolated striated muscles from medusa umbrella, muscle cells begin to aggregate. Aggregated striated muscles transdifferentiate into smooth muscle cells by the degradation of striated myofiber and the formation of flagella, which eventually form an endoderm and undergo morphogenesis into a manubrium.

Pioneering work by Schmid and colleagues has shown a transdifferentiation potential in jellyfish in vitro using the hydrozoan *Podocoryna carnea*. When isolated striated muscles from the medusa umbrella are cultured, the muscle cells form flagella and degrade myofibrils within 5–10 days, acquiring an intermediate character between striated muscle and smooth muscle [53]. After treatment with collagenase, the originally-striated muscle cells form a ball-shaped aggregate that can differentiate into seven to eight new cell types, which is similarly triggered after co-cultivation with umbrella endodermal cells (Figure 5) [54,55]. Intriguingly, the aggregate of isolates undergo morphogenesis to segregate the inner layer (endoderm), eventually self-organizing into a manubrium (Figure 5) [8,56]. Isolated striated muscles from other hydrozoan medusae also change cytoplasmic ultrastructure and form flagella, suggesting that muscle cell transdifferentiation may be a conserved feature in jellyfish [57]. Transdifferentiation processes followed by morphogenesis in *Podocoryna* likely include dedifferentiation into stem cells such that stem-like smooth muscle cells can self-replicate and differentiate into other cell types including specialized FMRFamide-positive nerve cells [58]. Indeed, while a striated muscle marker *Tpm2* is downregulated in isolated muscle cells, a stem cell marker *Cniwi* (the *Piwi* ortholog) is expressed in transdifferentiated cells [59]. Furthermore, *Msx*, a member of the muscle segment homeobox gene family, which is involved in dedifferentiation of mammalian myotubes [60], is expressed in transdifferentiated cells [61]. These results suggest that isolated striated muscle cells lose their identity and acquire stem-like characters before changing into different cell types.

Although detailed cell morphological changes have been described, it is currently unclear how trans/dedifferentiation in jellyfish is molecularly controlled. During the *Podocoryna* transdifferentiation, a basic bHLH transcription factor *Atl1* as well as *Bmp2/4* and *Bmp5/8* are upregulated in transdifferentiating cells [62,63]. bHLH family genes are involved in the self-renewal of neural progenitor cells and neuronal regeneration in planarians while BMP signaling controls cell cycle re-entry and dedifferentiation during newt limb regeneration [64,65]. It will be instructive to elucidate the roles of these molecules during trans/dedifferentiation in jellyfish.

Does cell fate plasticity mediated by trans/dedifferentiation contribute to jellyfish regeneration in vivo? One report describes a potential trans/dedifferentiation process during wound healing in the hydrozoan *Polyorchis penicillatus* [23]. When the *Polyorchis* subumbrella composed of striated muscle cells (myoepithelial cells) is partly removed, the surrounding myoepithelial cells migrate to the wound area by dramatically changing cellular morphology. In this process, myoepithelial cells lose polarity and myofibers, convert into migratory cells with lamellipodia protrusions, and eventually return to their original state as polarized myoepithelial cells, resembling the process of epithelial-to-mesenchymal transition and mesenchymal-to-epithelial transition during wound healing [66]. In order to confirm the cellular processes of trans/dedifferentiation in jellyfish in vivo, future studies should identify molecular markers for different cell types as well as introduce genetic lineage tracing methods.

## 5. Patterning

Regeneration relies not only on the reconstruction of lost structures by blastema formation but also requires organism-level patterning, or re-patterning, by remodeling the remaining parts and integrating the newly generated structure into the original body. For example, both re-patterning of body axes by positional control genes and remodeling of the existing organs (brain, gut, and pharynx) are essential for planarian regeneration [67]. *Hydra* also regenerate a functional body by reforming the head/foot organizer and the oral/aboral axis [68]. These body patterning events can occur even in small sized fragments of the body after amputation, enabling organisms to maintain physiological functions like moving and capturing food. For free-swimming medusae, patterning must include the re-establishment of radial symmetry that allows propulsion for swimming. This patterning was first reported in wild caught *Clytia* medusae wherein their umbrella fragments with various sizes and shapes returned to a typical circular shape within 12 h [12]. After circulation, each fragment regenerated its missing organs such as manubrium, tentacles, radial canals, and gonads, and finally became a functional organism, suggesting a whole-body regeneration of medusa.

**Figure 6:**
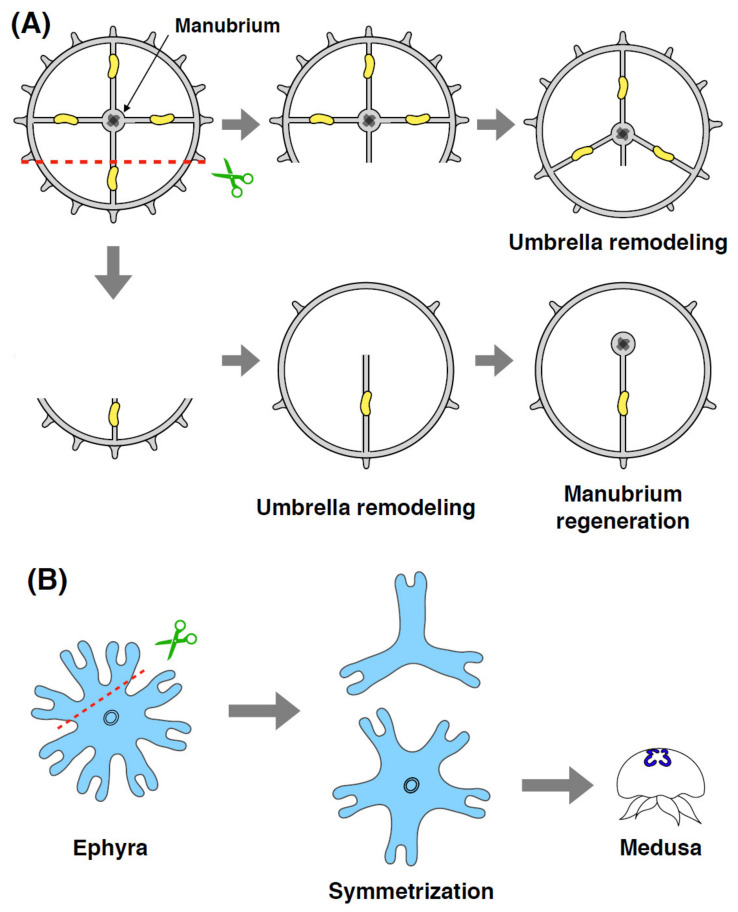
Schematic of patterning in jellyfish. (**A**) After dissection of the *Clytia* umbrella, different sizes of fragments undergo re-circularization and re-establish radial symmetry. Upon completion of umbrella remodeling, the manubrium can regenerate, even if the original manubrium is absent. (**B**) After amputation of *Aurelia* ephyra, the fragments repair their radial symmetry without regenerating lost arms, a phenomenon called “symmetrization”. Proper symmetrization of ephyra results in normal subsequent development into adult medusa if the oral part remains intact.

By utilizing systematic dissection experiments, Sinigaglia et al. have investigated patterning mechanisms in *Clytia* medusa [10]. When the umbrella is divided into fragments of different size, re-circularization of fragments occurs within 24 h (Figure 6A). In addition to phosphorylation of myosin regulatory light chain, actomyosin cables accumulate around the wound edge during re-circularization. Consistent with these observations, the myosin inhibitor blebbistatin suppresses re-circularization, suggesting that actomyosin activity is necessary for the remodeling of the umbrella. During umbrella remodeling, smooth muscle fibers are reconstructed and function as transient muscle hubs that predict the position of the regenerating manubrium. Intriguingly, transient muscle hubs either become stabilized or disappear depending on whether the manubrium remains in the fragment or not, allowing for the regeneration of organisms with only one manubrium (Figure 6A) [10]. Together, these results indicate that an actomyosin-driven mechanical force orchestrates the tissue remodeling underlying the re-establishment of radial symmetry, which is a prerequisite for subsequent organ regeneration in the appropriate place.

Although restoration of radial symmetry is required for whole-body regeneration in *Clytia* medusa, re-patterning events can occur without regeneration of lost body parts. The scyphozoan *Aurelia aurita* ephyra, a juvenile form of medusa, normally possesses eight radially symmetrical arms and exhibits swimming behaviors with pulsation. Upon amputation, *Aurelia* ephyrae immediately reconstruct radial symmetry with reduced numbers of arms rather than regenerating the missing body parts, a phenomenon referred to as ‘symmetrization’ (Figure 6B) [69]. The failure of symmetrization in ephyra results in a shrunken umbrella with abnormally large-sized manubrium in medusa, suggesting that symmetrization is a necessary process for normal development into adult. Interestingly, approximately 90% of the strobilation-derived ephyrae are octamers with eight arms while the remaining are non-octamers ranging from four to sixteen arms. Natural non-octamers develop normally and are morphologically indistinguishable from symmetrized ephyrae after amputation, indicating that symmetrization is an inherent mechanism that restores functional morphologies. Mechanistically, symmetrization does not involve cell proliferation or apoptosis, but it does require mechanical forces generated by muscle contraction during physiological pulsation [69]. Similar symmetrization in ephyrae is also observed after amputation in other scyphozoan species such as *Chrysaora pacifica*, *Mastigias* sp. and *Cotylorhiza tuberculate*. These results raise the possibility that symmetrization is a common self-repairing mechanism of scyphozoan ephyrae, which may have been acquired by responding to environmental stresses such as injury.

What are the molecular mechanisms that coordinate the patterning processes toward functional body regeneration? During *Clytia* umbrella remodeling, *Wnt6* is locally expressed in the remodeling edge and in the muscle hub [10]. Inhibition of actomyosin-driven remodeling by blebbistatin treatment suppresses *Wnt6* expression, suggesting that Wnt/β-catenin signaling is triggered by mechanical forces. In the regenerating *Hydra* polyp, Wnt/β-catenin signaling contributes to the re-patterning of the body axis as well as the remodeling of head and tail organizer [70,71]. Notably, actin fiber orientation is also involved in the reformation of the body axis during *Hydra* regeneration [72], implying a potential link of mechanical cues and Wnt/β-catenin signaling associated with re-patterning. Understanding how mechanical forces are converted into biochemical signals will be an important future avenue for regeneration research, which requires more examples from different taxa; jellyfish constitute a useful set of models for elucidating these underlying mechanisms.

## 6. Conclusions and Perspectives

Jellyfish regeneration involves a combination of cellular processes underlying wound healing, blastema formation, and systemic patterning that require organ or body-level communications. The evolutionally conserved wound closure mechanisms necessitate epithelial tissue repair upon injury. Cellular origins of renewing tissues and organs may derive from stem/progenitor cells and/or cell fate plasticity through trans/dedifferentiation of differentiated cells. While most cellular processes appear to be conserved across bilaterians and non-bilaterians, the reconstitution of radial symmetry may be a unique phenomenon in medusae, which will need further investigation in order to identify the mechanisms that coordinate repatterning events. Several important, but unanswered questions are as follows:

(1) *How do blastema and surrounding cells coordinate to generate lost organs?* During organ regeneration, stem/progenitor cells migrate to damaged sites and become a blastema for renewing tissue. *Clytia* medusae require a source of i-cells from the manubrium, gonads, or tentacle buds for regenerating a lost organ [10], suggesting the existence of mechanisms that convert multipotent i-cells into appropriate cell types for specific organs. It is unknown whether hydrozoan i-cells and non-hydrozoan amebocytes are heterogeneous populations or constitute a cellular hierarchy. Given that the potency of i-cells is different among hydrozoan species [33], it will be important to investigate cell potency of stem-like cells in jellyfish across different clades and within the same clade. Furthermore, it will be critical to decipher communications between stem-like cells and surrounding differentiated cells on the molecular level.

(2) *Are there any molecular landmarks that control radial symmetry during regeneration?* Positional control genes that belong to Wnt, BMP, or Hedgehog signaling and the *HOX* family have important roles in the development and maintenance of body axes in bilaterian [73,74,75,76]. In cnidarian polyps *Hydra* and *Nematostella*, these genes are necessary for pattering the body axes during development as well as for their repatterning during regeneration [77,78,79,80,81]. By contrast, the roles of most positional control genes during development and regeneration of the medusa body remain unclear. In particular, it will be illuminating to know how the same set of genes can control both developmental patterning and the reconstitution of radial symmetry that accompanies tissue remodeling.

(3) *What controls systemic regenerating responses?* While paracrine signaling from damaged tissue has been established to serve as local cues, the molecular framework that controls the systemic regenerative responses in jellyfish is unknown. Secreting factors such as neuropeptides and hormones may be involved in responses at distant sites by coordinating communications in different cell populations. It is also possible that the mechanical forces observed in umbrella remodeling and ephyra symmetrization account for global patterning entirely [10,69]. Furthermore, medusae possess organized nervous systems, nerve nets or nerve rings, but their roles in regeneration remain elusive. Given that nervous systems play an important role in organ regeneration in bilaterians [82], understanding their contribution in jellyfish regeneration will provide novel insight into the evolution of animal regeneration.

Addressing these questions will lead to a better understanding of the regeneration mechanisms associated with the reconstruction of the simple but elaborate organization of jellyfish morphologies. Considering the possibility that multiple cellular sources can become a potential blastema in different species, the regeneration potential of jellyfish must be diverse among medusozoans, and thus the following jellyfish species may serve as useful models for elucidating the diverse mechanisms of medusae regeneration:

(I) The scyphozoan moon jelly *Aurelia aurita* is one of the most familiar jellyfish in the world. The *Aurelia* medusa is relatively large in size (~30 cm) and can live in a wide range of temperatures from −1 to 32 °C [83]. In addition to accumulating evidence regarding *Aurelia* physiology and development [84,85], an assembled genome, transcriptome, and gene expression data set have been recently released [86,87]. As we have reviewed, *Aurelia* ephyra repair radial symmetry after injury [69]. Surprisingly, isolated medusa tentacles can transform into polyps [88,89], suggesting remarkable organismal level plasticity or reverse development. It is currently unknown to what extent adult *Aurelia* medusa can regenerate.

(II) The scyphozoan upside-down jellyfish *Cassiopea* has an unusual life style where medusa live upside-down and feed by releasing cassiosomes, or stinging-cell structures with mucus [90]. With the completion of the draft genome sequence for *Cassiopea xamachana* and relatively easy lab maintenance [91], *Cassiopea* will be a new jellyfish model for studying development, regeneration, and sleep-like behaviors [92]. Interestingly, the endosymbiotic dinoflagellate algae *Symbiodinium* lives with *Cassiopea* [93], providing a unique opportunity to study the impacts of endosymbiosis in jellyfish physiology and homeostasis. Previous studies have examined the regenerative capacities for different stages of *Cassiopea* [94,95,96]. A recent report further described that *Cassiopea* medusa can generate new ectopic sets of body structures after umbrella injury [97], suggesting a potential de novo whole-body regeneration from umbrella tissue.

(III) The cubozoan box jellyfish *Tripedalia cystophora* is characterized by a small body size (~1 cm) and specialized sensory structures, called rhopalia, located at the four corners of the medusa bell margin. Because the rhopalium is composed of six eyes that respond to light and visual stimuli, *Tripedalia* swim actively and avoid obstacles [98,99,100,101]. The eyes of *Tripedalia* are considered one of the most ancestral eyes with lenses in the animal kingdom, and they resemble the eyes of bilaterians in terms of structural and molecular features [102,103]. Importantly, *Tripedalia* can regenerate the functional rhopalium after injury [9]. The recent establishment of a transcriptome database will facilitate the use of *Tripedalia* for studying the development and regeneration of their complex sensory organ system [104].

(IV) The hydrozoan jellyfish *Clytia hemisphaerica* is an established model whose development has been well studied and whole life cycle can be robustly maintained in the laboratory [105,106]. In addition to the available genome and transcriptome resources, the recent advancement of gene editing by CRISPR/Cas9 and transgenesis enables genetic studies, which will provide a new opportunity to address different questions in jellyfish biology [107,108,109,110]. As introduced in this review, the *Clytia* medusa exhibit high regenerative abilities from the organ to the whole-body scale. In particular, whole body regeneration from fragments is a fascinating system. The relatively small size and transparent body with amenable genetic tools allow identification of regeneration mechanisms at the cellular and molecular levels. Further investigation of stem-like cells will reveal their role and potency during development and regeneration.

(V) The hydrozoan jellyfish *Cladonema* (*Cladonema pacificum* or *Cladonema radiatum*) is characterized by branched tentacles and eye-like photoreceptor organs called ocelli in the medusa stage (Figure 2B) [111,112]. The small-sized medusa (~1 cm) is benthic and can be kept in the lab without any special equipment. In addition, spawning is controlled by light-dark transitions [113,114], which together enable easy lab maintenance. Consistent with regenerative capacities described in other hydrozoans, the *Cladonema* medusa regenerate multiple organs including tentacles and ocelli [8,11]. Furthermore, isolated medusa buds from polyps can transform into stolons and/or polyps [115], suggesting reverse development potential. By introducing genetic manipulations and establishing genome and transcriptome resources, *Cladonema* will be a powerful model for studying mechanisms of regeneration.

In summary, jellyfish regenerate relatively complex organs and whole bodies by orchestrating diverse cellular dynamics and cell/tissue communications. Reconstitution of radial symmetry is likely a key step for regenerating functional organs and organisms. Given that medusae have acquired more complex morphology than polyps during evolution, it is tempting to compare regeneration mechanisms between polyps and medusae among different species and within the same species, which will shed light on how regeneration potential has diversified in cnidarians as well as across medusozoans. Furthermore, the reverse development phenomenon from medusae to polyps will provide a new research paradigm to address mechanisms of potential rejuvenation. The recent advancement of sequencing technology combined with new genetic tools will facilitate the use of model jellyfish or even integrate diverse jellyfish as new models for addressing many unanswered questions, which together will open up a new horizon of regeneration studies.

## Data Availability

Not applicable.
